# Development and application of loop-mediated isothermal amplification for detection of the F167Y mutation of carbendazim-resistant isolates in *Fusarium graminearum*

**DOI:** 10.1038/srep07094

**Published:** 2014-11-18

**Authors:** Yabing Duan, Xiaoke Zhang, Changyan Ge, Yong Wang, Junhong Cao, Xiaojing Jia, Jianxin Wang, Mingguo Zhou

**Affiliations:** 1 College of Plant Protection, Nanjing Agricultural University, Nanjing, 210095, China

## Abstract

Resistance of *Fusarium graminearum* to carbendazim is caused by point mutations in the *β*_2_-tubulin gene. The point mutation at codon 167 (TTT → TAT, F167Y) occurs in more than 90% of field resistant isolates in China. To establish a suitable method for rapid detection of the F167Y mutation in *F. graminearum*, an efficient and simple method with high specificity was developed based on loop-mediated isothermal amplification (LAMP). A set of four primers was designed and optimized to specially distinguish the F167Y mutation genotype. The LAMP reaction was optimal at 63°C for 60 min. When hydroxynaphthol blue dye (HNB) was added prior to amplification, samples with DNA of the F167Y mutation developed a characteristic sky blue color after the reaction but those without DNA or with different DNA did not. Results of HNB staining method were reconfirmed by gel electrophoresis. The developed LAMP had good specificity, stability and repeatability and was suitable for monitoring carbendazim-resistance populations of *F. graminearum* in agricultural production.

Fusarium head blight (FHB) or scab, caused mainly by *Fusarium graminearum* (teleomorph = *Gibberella zeae*), is one of the most common fungal diseases of cereal crops worldwide^1^,^2^. Under favorable conditions, this fungus can cause severe yield losses and produce mycotoxins that are harmful to humans and animals^3^,^4^,^5^,^6^,^7^,^8^. Owing to low levels of host resistance, application of fungicides is the principal method for controlling FHB^9^,^10^. Carbendazim and other benzimidazole fungicides with the action mode of mitosis inhibition, have been shown to be effective against a variety of plant pathogenic fungi, including most ascomycetes and some deuteromycetes, and also used to control FHB over the past three decades^11^. Carbendazim and its mixtures are still the main reagents for controlling FHB in China. However, carbendazim-resistant *Fusarium* populations have increased under high selective pressure of fungicide in recent years, and FHB in China has become more severe^12^,^13^,^14^.

Previous studies on the resistance of *F. graminearum* to carbendazim is mainly caused by point mutations in the *β*_2_-tubulin gene (FGSG 06611.3)^11^,^15^,^16^,^17^,^18^, including codons 50 (TAC → TGC, Y50C), 167 (TTT → TAT, F167Y), 198 (GAG → CTG, E198L; GAG → CAG, E198K; GAG → AAG, E198Q) and 200 (TTC → TAC, F200Y). Among the carbendazim-resistant isolates from the field, the mutation genotype at codon F167Y was dominant, accounting for more than 90% of the resistant isolates^15^.

In order to predict the development of fungicide resistance and to use fungicide and other disease control tactics effectively, researchers must develop methods for resistance early detection. Previous detection methods have been established by mycelial growth inhibition and spore germination assay on PDA containing the different concentrations^13^,^19^, but the procedure is time-consuming and tedious, and the detection efficiency is low. Several common techniques were developed in recent years based on single nucleotide polymorphisms (SNPs), such as DNA sequencing, allele-specific PCR (ASPCR)^20^, single-strand conformation polymorphism PCR (SSCP-PCR)^21^, the mismatch amplification mutation assay (MAMA-PCR)^22^, denaturing high-performance liquid chromatography (DHPLC)^23^, PCR-restriction fragment length polymorphism (PCR-RFLP)^24^, TaqMan-MGB^25^, the LightCycler-based PCR hybridisation mutation assay^26^ and primer-introduced restriction analysis PCR (PIRA-PCR)^4^. DNA sequencing is the standard method for identifying mutations, but it requires much more time and is expensive when a large number of samples must be analyzed. Although ASPCR is an excellent method, its use frequently requires extensive optimisation, and background amplification is often high. PCR-RFLP is based on the alteration of a restriction enzyme site caused by a point mutation, so it is useless when the point mutation does not result in the alteration of a restriction enzyme site. Based on the point mutation at codon F167Y (TTT → TAT, F167Y) of the *β*_2_-tubulin gene in *F. graminearum*, PIRA-PCR and cycleave PCR methods were developed for rapid detection of *F. graminearum* genotypes (F167Y) resistant to carbendazim^4^,^17^. The methods could detect carbendazim resistance much more rapidly than the classical mycelial growth inhibition and spore germination assays. However, they have several intrinsic disadvantages, including the requirement of rapid thermal cycling, insufficient specificity, and rather low amplification efficiency^4^,^17^. Herein, we developed a new nucleic acid amplification method that can be used to detect the resistant mutants of *F. graminearum* to the carbendazim-group fungicides in the absence of a thermal cycler in this study.

Loop-mediated isothermal amplification (LAMP), a novel technique, has been developed, which can amplify nucleic acids with high specificity, sensitivity and rapidity under isothermal conditions, and employ self-recurring strand-displacement synthesis primed by a specially designed set of target-specific primers^27^. A set of four specially designed primers, which recognize a total of six distinct sequences on the target, was used for the LAMP assay. An inner primer containing sequences of the sense and antisense strands of the target DNA initiates LAMP. The following strand-displacement DNA synthesis primed by an outer primer releases a single-stranded DNA. This serves as a template for DNA synthesis primed by the second inner and outer primers that hybridize to the other end of the target, which produces a stem-loop DNA structure. In the subsequent LAMP cycling, one inner primer hybridizes to the loop on the product and initiates displacement DNA synthesis, yielding the original stem-loop DNA and a new stem-loop DNA with a stem twice as long. The cycling reaction continues with accumulation of 10^9^ copies of target in less than an hour. The final products are stem-loop DNAs with several inverted repeats of the target and cauliflower-like structures with multiple loops formed by annealing between alternately inverted repeats of the target in the same strand^28^,^29^,^30^. LAMP products can be visualized with naked eyes by adding DNA-intercalating dyes such as ethidium bromide, SYBR Green I, propidium iodide, or Quant-iTPicoGreen; by adding metal-ion indicators such as hydroxynaphthol blue (HNB)^31^, CuSO_4_^32^, or calcein^33^ or by measuring the increase in turbidity derived from magnesium pyrophosphate formation (to infer increases in amplified DNA concentration). LAMP products can also be detected by real-time detection methods^34^. The simplicity of the LAMP method, which does not require a thermal cycler, makes it suitable for detecting field resistant populations.

During the last 10 years, with the development of LAMP, it has been widely applied in qualitative detection of many plant pathogenic microorganisms, such as *Phytophthora sojae*^35^, *F. graminearum*^36^, *Sclerotinia sclerotiorum*^37^, *Meloidogyne enterolobii*^38^, *Botrytis cinerea*^39^, and phytoplasmas^40^. However, the application of LAMP on the field of fungicide resistance has not been reported. We developed a LAMP assay for the detection of carbendazim-resistant isolates of *F. graminearum* based on the point mutation at codon 167 of the *β*_2_-tubulin gene and demonstrated that the assay was applicative and efficient in fields. The new LAMP assay will be valuable to cereal growers because it will help guide the management of carbendazim resistance in FHB timely.

## Results

### Optimization of LAMP primers

**Figure 1:**
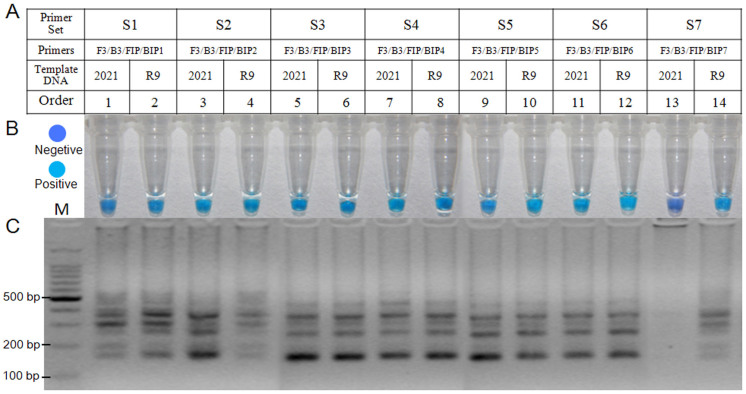
Optimization of LAMP primers. (A) Information for LAMP primer sets used for optimization. (B) Assessment was based on HNB visualization of color change of the LAMP products. (C) Assessment was based on gel electrophoresis analysis of the LAMP products.

Based on the point mutation (TTT → TAT, F167Y) of the *β_2_*-tubulin gene of carbendazim -resistant mutant R9, a set of LAMP primers was designed using the Primer explorer V4 software program. LAMP is very sensitive, and it is occasionally possible for products to be amplified from the wild types; thus, one or two mismatches were added to a primer in each primer set. The mismatches were added to increase the likelihood that the primers would specifically amplify the F167Y substitution. Seven sets of LAMP primers (Table S1) were tested to identify appropriate primers using the genomic DNA of the wild type isolate 2021 and the F167Y mutant R9. As shown in Fig. 1, only a set of LAMP primers (S7) was appropriate for detecting the mutation. The positive LAMP reaction was indicated by a sky blue color; while the negative reaction remained violet color (Fig. 1B). After the tubes were visually assessed by color change, they were analyzed by gel electrophoresis. As expected, the typical ladder-like pattern on 3.0% gel electrophoresis was observed in a positive sample, but not in the negative control (Fig. 1C). The results showed that the primer set S7 (Fig. 1, Table S1) could be used to distinguish the F167Y mutation of *F. graminearum*.

### Optimization of LAMP reaction

**Figure 2:**
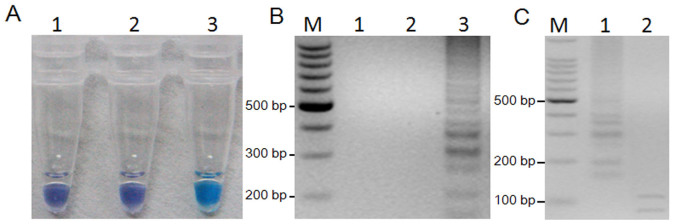
LAMP detection of the F167Y mutation in *F. graminearum* and digestion of positive LAMP products. (A) LAMP for detection of the F167Y mutation using HNB as a visual indicator. The reaction becomes sky blue if the *β_2_*-tubulin gene has a point mutation at codon F167Y but remains violet if the *β_2_*-tubulin gene has no mutation or other mutation at codon F167Y. 1, ddH_2_O; 2, 2021; 3, R9. (B) Agarose gel electrophoresis of LAMP products. The positive reaction is manifested as a ladder-like pattern on the 3.0% agarose gel. M, 100-bp ladder; 1, ddH_2_O; 2, 2021; 3, R9. (C), LAMP products were digested with Pvu I, and two fragments (119 bp, 88 bp) were observed by 3.0% agarose gel. M, 100-bp ladder, 1, LAMP products without digestion; 2, LAMP products digested by Pvu I.

When LAMP was performed with the mutant R9 DNA as the template, the best results were obtained in a 25 μL volume containing 6 U of Bst DNA polymerase, 2.5 μL 10 × ThermoPol Buffer, 4 mM MgCl_2_, 1 mMdNTPs, 1.6 μM each of FIP and BIP, 0.2 μM each of F3 and B3, 0.94 M betaine, 150 μM HNB, and 1 μL of target DNA. The reactions were performed in 0.2-mL centrifuge tubes in a water bath for temperature control. As expected, the typical ladder-like pattern on 3% gel electrophoresis (Fig. 2B) and visual detection with HNB (Fig. 2A) were observed in the mutant R9 but not in the wild type isolate 2021 and ddH_2_O.

### Optimization of LAMP reaction conditions

**Figure 3:**
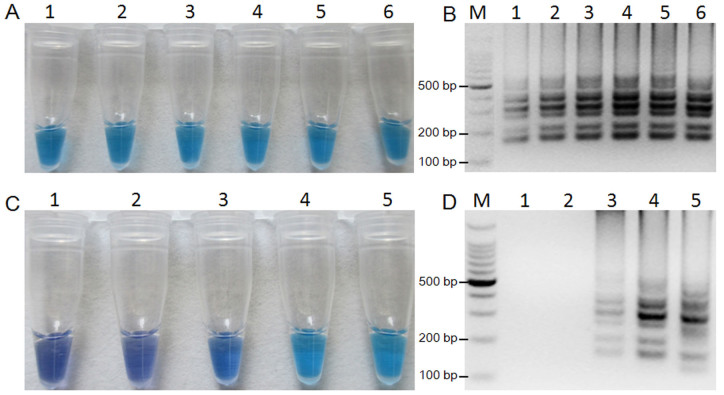
Optimization of LAMP reaction temperature (A, B) and reaction time (C, D). Assessment was based on HNB visualization of color change in (A) and (C) and on gel electrophoresis in (B) and (D). M = 100-bp ladder. In (A) and (B), 1 = 60°C, 2 = 61°C, 3 = 62°C, 4 = 63°C, 5 = 64°C, and 6 = 65°C. In (C) and (D), 1 = 15 min, 2 = 30 min, 3 = 45 min, 4 = 60 min, and 5 = 90 min.

With the reaction reagents optimized as previously indicated, LAMP was performed using the mutant R9 DNA as template to determine the optimal reaction temperature and time. Color change at different temperatures was consistent (Fig. 3A); however, the intensity of DNA at 63°C was the strongest among all of the test temperatures (Fig. 3B). When LAMP was performed at 63°C with a range of test time, positive results were obtained in HNB-visualization (Fig. 3C) or gel electrophoresis (Fig. 3D) from 45 to 90 min, while the ladder-like pattern produced by gel electrophoresis was the strongest at 60 min. Therefore, the optimal reaction condition of LAMP for the F167Y mutation types was 63°C for 60 min.

### Sequencing and restriction endonuclease digestion of LAMP products

As shown in Fig. 2B, positive LAMP products showed a ladder-like pattern on gel electrophoresis. In addition, LAMP products could be observed by naked eyes under natural light. The color changed from violet to sky blue in the positive amplification (Fig. 2A). Sequence analysis was determined to further confirm the LAMP products. The recombinant plasmid *pEASY*®-T1-N207 indicated that the 207 bp target sequence was 100% homologous to the sequence of the *β_2_*-tubulin gene used for the primers design. After digestion of LAMP products with the Pvu I, the 88 and 119 bp fragments were observed (Fig. 2C), and were in good accordance with those predicted theoretically from the expected structures. The results of the sequence and the digestion confirmed that the products were amplified specifically from the *β_2_*-tubulin target region in *F. graminearum*.

### Specificity of the LAMP assay

**Figure 4:**
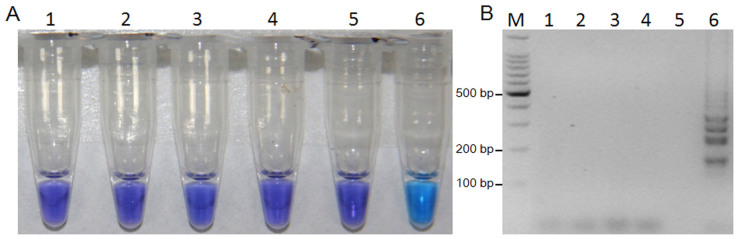
Specificity of LAMP detection of the F167Y mutation in *F. graminearum*. Assessment was based on (A) HNB visualization of color change or (B) gel electrophoresis analysis of the LAMP products. M, a 100-bp ladder; 1, 2021; 2, Y50C; 3, J-2; 4, ZJ80; 5, NT-7; 6, R9.

The LAMP assay was positive only for the mutant R9, no positive DNA products were observed when other mutants or isolates (Table S2) were used as templates. This was true when assessment was based on HNB-visualization (Fig. 4A) or gel electrophoresis (Fig. 4B).

### Repeatability of the LAMP assay

The robustness and repeatability of the LAMP method were tested with 28 known F167Y mutants from different geographical regions (Table S3). All the mutants were positive based on HNB-visualization or gel electrophoresis. It suggested that the established LAMP assay had good repeatability and stability.

### Evaluation of LAMP using the perithecia produced on rice stubbles in the field

To demonstrate the applicability of the LAMP assay in field samples, 116 rice stubbles with perithecia were tested by the LAMP assay and the MIC method. The positive-sample ratios were 41.38% and 43.97% by the LAMP assay and MIC method (Table 1), respectively. Additionally, the resistant mutation genotypes were confirmed by sequencing, which was consistent with the MIC method. All the resistant mutants (F167Y) were positive based on HNB-visualization or gel electrophoresis, whereas other mutants and wild type strains were negative. This showed that the LAMP assay could successfully detect the F167Y mutation genotype in the perithecia produced on rice stubbles in field.

### Application of LAMP on monitoring carbendazim-resistance populations of *F. graminearum* in agricultural production

The aim of these experiments was to demonstrate the application of LAMP on resistance monitoring of *F. graminearum* to carbendazim in agricultural production. In 2013, 340 isolates from infected wheat spikelets were tested by MIC and the ratio of resistant mutants was 41.47%. To further verify the application of LAMP on resistance monitoring, LAMP was also performed using DNA of these isolates as templates, the positive-sample ratio was 38.82% (Table 2). The resistance ratio of the F167Y mutation genotype in all the resistant mutants was 93.62%.

In 2014, 1894 infected wheat spikelets were tested by LAMP and MIC. The positive-sample ratios were 497/1894 (26.24%) by LAMP and 519/1894 (27.4%) by MIC (Table 3). The positive-sample ratio by LAMP was up to 95.76% in all the resistant mutants. This result suggested that LAMP was very feasible to detect the resistant mutants in field. Therefore, LAMP reported here might be used for monitoring carbendazim-resistance populations of *F. graminearum* in agricultural production.

## Discussion

LAMP is a novel nucleic acid amplication technique, which amplifies with high specificity, sensitivity, and rapidity under isothermal conditions. During the last 10 years, due to rapid amplification, simple operation, and easy detection, LAMP has been successfully applied in pathogenic microorganisms^28^. However, it has not been used to detect mutations causing target-site-mediated fungicide resistance in plant pathogens. In this study, a highly practical and valid method for the detection of the F167Y mutation in *F. graminearum* was developed. To the best of our knowledge, this is the first report on the application of the LAMP assay for monitoring fungicide resistance in plant pathogens.

To distinguish the F167Y mutation from the wild type, the mismatched primers were designed using the Primer Explorer V4 software program based on the point mutation (TTT → TAT, F167Y) of the *β_2_*-tubulin gene of MBC-resistant mutant R9. However, it will be a challenge to identify the best set of primers among several possibilities. In this study, seven primer sets were tested, and only S7 was appropriate for detecting the mutation (Fig. 1). Nagamine et al.^30^ accelerated the LAMP reaction by loop primers, which suggested that the LAMP reaction times would be shorter than the original method when using loop primers. With the primer set S7, suitable loop primers were identified and used to accelerate the reaction. However, the established LAMP assay could not distinguish the F167Y mutation from the wild type when using the loop primers. Based on the manual for LAMP primer designing, the loop primers are not essential for LAMP, so the LAMP reaction was performed without loop primers.

Based on the selected best primer set (S7), the concentrations of reaction components and reaction conditions of LAMP were optimized and the LAMP assay was developed in this study. As the LAMP reaction progresses, pyrophosphate ions are produced, which bind to Mg^2+^ ions and form a white precipitate of magnesium pyrophosphate. Therefore, the results of the LAMP can be visualized with the naked eyes by adding metal-ion indicators such as hydroxynaphthol blue (HNB)^31^, CuSO_4_^32^, or calcein^33^. Moreover, LAMP products can also be judged with the naked eyes by adding DNA intercalating dyes, or by measuring the increase in turbidity derived from magnesium pyrophosphate. DNA intercalating dyes such as SYBR green^41^ or Picogreen^42^ can be added after a reaction is completed. Due to the final addition of these intercalating dyes and exposed operation, most of the available methods suffer from increasing the rate of contamination^27^,^33^,^43^,^44^. To avoid this, HNB is added before incubation so that amplification is completed in a closed tube system, and the detection of color change requires no equipment. A positive reaction is indicated by a color change from violet to sky blue, and a negative reaction retains violet. In the current study, the positive and negative reactions could be successfully distinguished with HNB, and confirmed when the LAMP products were subjected to gel electrophoresis analysis.

Compared with conventional detection methods, LAMP assay is easier to perform and more rapid, and the results are easier to evaluate. LAMP operates under isothermal conditions and the optimal temperature was set as 63°C. LAMP is also rapid and 60 min was optimal for detection of the F167Y mutation in *F. graminearum*. Because LAMP is conducted isothermally, the thermal cycling required in PCR is unnecessary. Moreover, LAMP requires only a regular laboratory bath or heat block that can provide a constant temperature of 63°C. It is simple and should be useful even for those laboratories and research institutes unfamiliar with PCR or other methods of molecular analysis.

To confirm the efficiency and specificity of LAMP, DNA extracted from the mutant R9 (F167Y) and other mutants or wild-type isolates (Table S2) were used as templates for LAMP assay. The LAMP assay could distinguish the F167Y mutation specifically in *F. graminearum*. Restriction enzyme and sequence analyses also validated its specificity (Fig. 2C).

To determine the robustness and repeatability of LAMP, 28 known F167Y mutants from the geographical regions were tested. All the samples were positive with LAMP, and amplicons were confirmed through 3% gel electrophoresis. Meanwhile, the sensitivity of LAMP and PCR was tested using 10-fold serial dilutions of genomic DNA. The developed LAMP method had ten-fold higher sensitivity than that of the conventional PCR (Fig. S1). These results indicated that the LAMP assay established in this study had good repeatability and sensitivity.

In the fields, the ascospores from the fruiting bodies perithecia on rice stubbles are the primary infection source of FHB^40^. To determine the applicability of LAMP in field samples, 116 rice stubbles with perithecia were tested by LAMP and MIC. The *β_2_*-tubulin gene of the resistant mutants by the MIC was amplified and sequenced. Sequencing result showed that all the positive samples by LAMP had a point mutation at codon 167 of the *β_2_*-tubulin gene. The ratio of the F167Y mutation in all the resistant mutants was 48/51 (94.12%), which was also confirmed by sequencing. Therefore, the LAMP assay could successfully detect the F167Y mutation in the perithecia produced on rice stubbles. This will be used for early warning of resistance risk in *F. graminearum* to MBC in the field. Moreover, this will also provide important reference information for control tactics of FHB. In this study, we also demonstrated the application of LAMP on monitoring MBC-resistance populations of *F. graminearum* in agricultural production. In 2013, the resistance frequency by MIC and LAMP was 41.47% and 38.82%, respectively. In 2014, the resistance frequency by MIC and LAMP was 27.4% and 26.24%, respectively. Compared with other methods, the newly developed LAMP significantly improved the detection efficiency. High resistance frequency will lead to control failures. Based on the data of carbendazim-resistance monitoring during 2013–2014, the use of carbendazim in controlling FHB should be reduced. Therefore, the LAMP method developed in this study was very feasible to detect the resistant mutants in the field.

In conclusion, A LAMP assay combined with HNB was established and demonstrated to be more sensitive, specific, and practical for detection of the F167Y mutation in *F. graminearum* than previous methods. Therefore, it will be potentially useful for monitoring and management of carbendazim resistance in *F. graminearum* in the future.

## Methods

### Fungal isolates and reagents

*Fusarium graminearum* isolate R9 (Table S2), which is resistant to MBC because of a point mutation at codon 167 (Phe to Tyr, F167Y) in the *β*_2_-tubulin gene (Genbank accession no. FJ214663), and *F. graminearum* isolate 2021 (Table S2), which is sensitive to MBC, were used in this study. Other *F. graminearum* isolates were collected from diseased wheat spikelets from different geographical regions in China (Table 2, 3).

Carbendazim was provided by the Shenyang Academy of Chemistry and Industry, China. The fungicide was dissolved in 0.1 M hydrochloric acid (HCl) and adjusted to 1 mg mL^−1^. Bst DNA polymerase was purchased from NEB. Betaine and hydroxynaphthol blue (HNB) were purchased from Sigma, and MgCl_2_ and dNTPs were purchased from Takara. Double-distilled water (ddH_2_O) was used in all experiments. All other reagents were analytical grade.

### Culture conditions and DNA extraction

Autoclaved potato dextrose agar (PDA) was used to culture the fungi and in routine assays for sensitivity to carbendazim *in vitro*. Based on differences in carbendazim sensitivity, tested strains were divided into four phenotypes according to the minimum inhibitory concentration (MIC)^18^ method as follows: high carbendazim resistance, MIC > 100 μg mL^−1^; moderate carbendazim resistance, 100 μg mL^−1^ > MIC > 25 μg mL^−1^; low carbendazim resistance, 25 μg mL^−1^ > MIC > 1.4 μg mL^−1^; carbendazim sensitive, MIC < 1.4 μg mL^−1^. Genomic DNA of the isolates 2021 and R9 were extracted using the Plant Genomic DNA Kit (Tiangen) according to the manufacturer's instructions. Genomic DNA of other isolates was extracted using the CTAB method^45^.

### Primer design

**Figure 5:**
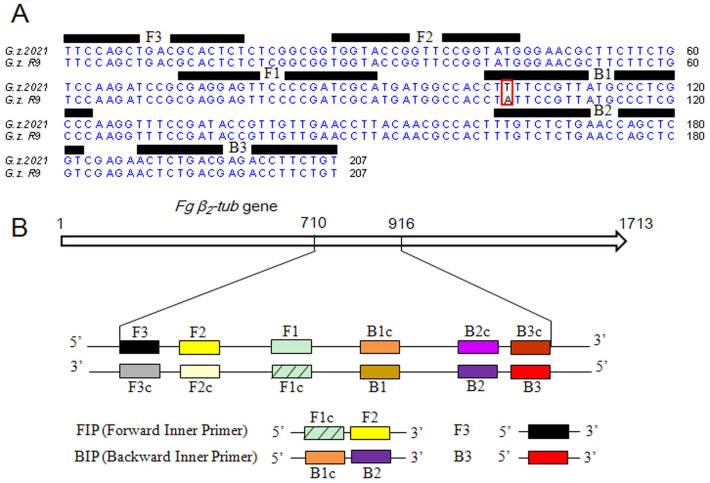
Design of LAMP primers for detection of the F167Y mutation in *F. graminearum*. (A) Nucleotide sequence alignment of the target region *β_2_-tub* in wild-type isolate 2021 and MBC-resistant isolate R9. The sequences used for LAMP primers are indicated by bold lines. One point mutation in red frame (TTT → TAT) leads to a moderate level of MBC resistance. (B) Schematic representation of the LAMP primers used in this study. Construction of the inner primers FIP and BIP are shown. F1c and B1c are complementary to F1 and B1, respectively.

PCR primers (Table S1) were designed using Primer Premier 5.0 (Premier, Canada) and Oligo 6.0 (MBI, Cascade, CO) software. Four specific LAMP primers were designed based on the *β_2_*-tubulin gene of *F. graminearum* (FGSG 06611.3, http://www.broadinstitute.org), using the Primer explorer V4 software program (http://primerexplorer.jp/e/). The structure of the LAMP primers and their complementarity to target DNA used in this study are shown in Fig. 5. A forward inner primer (FIP) consisted of F1c and F2, and a backward inner primer (BIP) consisted of B1c and B2. The outer primers F3 and B3 were required for initiation of the LAMP reaction. Primer pair Fgbeta637F/Fgbeta637R was used for conventional PCR of the *β_2_*-tubulin gene of *F. graminearum*. Information regarding the primer names and sequences are provided in Table S1.

### Optimization of LAMP primers

To distinguish the *F. graminearum* genotypes (F167Y) from the wild types, the mismatched primers (Table S1, Fig. 5) were designed and optimized to specially amplify the mutation genotypes (F167Y), but not the wild type. The LAMP assay was performed in 0.2-mL micro centrifuge tubes using the genomic DNA of R9 as a template, DNA of the wild type 2021 and ddH_2_O as negative control. The reaction tubes were placed in a water bath for 60 min at 65°C and then heated for 10 min at 80°C to terminate the reaction. Each product was analyzed by 3.0% agarose gel electrophoresis stained with ethidium bromide and photographed under a UV transilluminator. In addition, the amplification product could also be visually inspected by naked eyes according to the color change from violet to sky blue, while the negative control remained violet. There were four replications for each treatment, and the experiment was repeated twice.

### Optimization of LAMP reaction

The LAMP reaction was performed in a total volume of 25 μL. For the optimization of reagents, a range of concentrations of Bst DNA polymerase large fragments (0.16–0.64 U μL^−1^), dNTPs (0.2–2 mM), Mg^2+^ (2–8 mM), primers (0.2–2 μM), betaine (0.8–1.6 M), and HNB (100–200 μM) were evaluated. The LAMP assay was done as described earlier. As before, the assays were assessed based on HNB-visualized color change and gel electrophoresis. Each treatment had three replications, and the experiment was performed three times.

### Optimization of LAMP reaction conditions

The LAMP reaction mixtures were incubated for 45 min at 60, 61, 62, 63, 64, or 65°C to determine the optimal reaction temperature. Then, the LAMP was performed at the optimal reaction temperature for 15, 30, 45, 60, and 90 min to determine the optimal reaction time. The reactions were terminated by heat inactivation at 80°C for 10 min. The assays were assessed based on HNB-visualized color change and then on gel electrophoresis as described in the previous section. There were three replicates for each treatment, and the experiment was repeated twice.

### Sequencing of LAMP products

After electrophoresis, 207-bp DNA bands obtained from the positive LAMP reaction on gel were extracted using a Gel Extraction Kit (Omega, USA), and were amplified by PCR using the primers F3 and B3. PCR reaction mixtures contained 10 μM F3 and B3 (2 μL each primer), 2.5 mM dNTPs (2 μL), 10 × PCR buffer (Mg^2+^ Free, 2.5 μL), 25 mM MgCl_2_ (1.5 μL), 5 U μL^−1^ rTaq (0.125 μL), template DNA (1 μL), and ddH_2_O (13.875 μL). PCR reactions were performed as follows: 94°C for 2 min, and then 35 cycles of denaturation at 94°C for 30 s, annealing at 54°C for 30 s, extension at 72°C for 30 s, with a final extension at 72°C for 10 min. The 207-bp product was extracted after 1% agarose gel electrophoresis, cloned into *pEASY*®-T1 Cloning Vector (Transgen, Beijing), and then transformed into competent TOP10 cells. The recombinant plasmid *pEASY*®-T1-N207 was extracted from positive clones and sequenced by Sangon (China).

### Restriction enzyme digestion analysis of LAMP products

LAMP products were digested with Pvu I in a 20 μL reaction containing Pvu I (1 μL) (Takara), 10 × K Buffer (2 μL), 0.1% BSA (2 μL), LAMP products (8 μL), and ddH_2_O (7 μL), incubated at 37°C overnight, after which the DNA bands were analyzed on 3% agarose gel electrophoresis stained with ethidium bromide and photographed as above.

### LAMP specificity test

LAMP specificity was verified by performing the LAMP assay of DNA of the wild type isolate 2021, carbendazim-resistant mutant R9 and other carbendazim-resistant mutants of *F. graminearum* (Table S2). The LAMP assay was performed and assessed as described in the previous section. The test was performed three times with three replications.

### LAMP repeatability test

To evaluate the accuracy of LAMP, the known *F. graminearum* (F167Y) mutants (n = 28) from diseased spikelets from different geographical regions in China were tested with ddH_2_O as negative control under the same condition (Table S3).

### Evaluation of LAMP using the perithecia produced on rice stubbles in field

To evaluate the feasibility of LAMP in field, 116 rice stubbles with perithecia collected from different fields in Jiangsu Province of China (Table 1) were tested by LAMP as described above. The single pile of perithecia was divided into two equal groups. One group was transferred to a 2-mL centrifuge tube, and ground using the tissue lyser (MM400, Retsh). DNA was extracted using the CTAB method^45^ and used as the template of LAMP. The other group was also tested using the MIC method described above. The primers Fgbeta637F/Fgbeta637R (Table S1) were used to amplify the *β_2_*-tubulin gene of the positive samples by the MIC method. PCR reactions were performed as described above. The 637-bp product was extracted after gel electrophoresis, cloned into *pEASY*®-T1 Cloning Vector (Transgen, Beijing), and then transformed into competent TOP10 cells. The recombinant plasmid *pEASY*®-T1-637 was extracted from positive clones and sequenced by Sangon (China).

### Application of LAMP on monitoring carbendazim-resistance populations of *F. graminearum* in agricultural production

In 2013, a total of 340 isolates were collected from infected wheat spikelets from different fields in Jiangsu province of China (Table 2). To determine the sensitivity of *F. graminearum* to carbendazim, these isolates were placed on PDA plates containing 5 μg mL^−1^ carbendazim. After the plates had been incubated at 25°C for 5 days, the isolates which could grow were resistant. To further verify the application of LAMP on detecting resistant mutants (F167Y), DNA of these resistant mutants was extracted and LAMP was performed as described above.

To directly demonstrate the application of LAMP on resistance monitoring of *F. graminearum* to carbendazim in agricultural production, a total of 1894 isolates were collected from infected wheat spikelets from different provinces of China in 2014 (Table 3). These infected wheat spikelets were divided into two equal groups. One group was tested by LAMP and the other group was tested using MIC method.

## Author Contributions

Conceived and designed the experiments: Y.B.D. and C.Y.G. Performed the experiments: Y.B.D., X.K.Z., C.Y.G., Y.W., J.H.C. and X.J.J. Analyzed the data: Y.B.D., C.Y.G., J.H.C. and J.X.W. Wrote the paper: Y.B.D. Revised and approved the final version of the paper: M.G.Z.

## Supplementary Material

Supplementary Information Supplementary data

## Figures and Tables

**Table 1:** Rice stubbles used in this study, numbers that were positive in LAMP and MIC, and numbers of the different mutation genotypes

				Number of the mutation genotypes		
Geographical origin	Number of samples	Positive in LAMP	Positive in MIC	F167Y	F200Y	E198L
Nanjing, Jiangsu	18	5	5	5	0	0
Yancheng, Jiangsu	15	8	9	8	1	0
Huai'an, Jiangsu	33	16	16	16	0	0
Nantong, Jiangsu	11	3	4	3	1	0
Taizhou, Jiangsu	21	8	9	8	0	1
Zhenjiang, Jiangsu	18	8	8	8	0	0
Total	116	48	51	48	2	1

**Table 2:** Infected wheat spikelets used in this study, numbers that were positive in LAMP and MIC, and resistance frequency obtained from LAMP and MIC in 2013

Geographical origin	Number of samples	Positive in LAMP	Resistance frequency by LAMP (%)	Positive in MIC	Resistance frequency by MIC (%)
Zaonan, Jiangsu	102	42	41.18	46	45.1
Shiyan, Jiangsu	37	16	43.24	16	43.24
Hong'an, Jiangsu	34	9	26.47	10	29.41
Jiangliu, Jiangsu	87	29	33.33	29	33.33
Guanghui, Jiangsu	42	12	28.57	15	35.71
Shahe, Jiangsu	38	24	63.16	25	65.79
Total	340	132	38.82	141	41.47

**Table 3:** Infected wheat spikelets used in this study, numbers that were positive in LAMP and MIC, and resistance frequency obtained from LAMP and MIC in 2014

Geographical origin	Number of samples	Positive in LAMP	Resistance frequency by LAMP (%)	Positive in MIC	Resistance frequency by MIC (%)
Zaoyang, Hubei	84	0	0	0	0
Yuncheng, Shanxi	78	0	0	0	0
Xiaoxian, Anhui	114	0	0	0	0
Jingzhou, Hubei	75	0	0	0	0
Lujiang, Anhui	75	11	14.67	13	17.33
Yanggu, Shandong	140	0	0	0	0
Xihua, Henan	75	0	0	0	0
Yandu, Jiangsu	79	31	39.24	31	39.24
Binhai, Jiangsu	231	54	23.38	57	24.68
Dafeng, Jiangsu	200	96	48.0	107	53.5
Xiangshui, Jiangsu	294	71	24.15	72	24.49
Jianhu, Jiangsu	260	143	55.0	157	60.38
Dongtai, Jiangsu	189	76	40.21	82	43.39
Total	1894	497	26.24	519	27.4
