# Supplementary material for: Development and application of loop-mediated isothermal amplification for detection of the F167Y mutation of carbendazim-resistant isolates in Fusarium graminearum
Source: Sci Rep. 2014 Nov 18;4:7094. doi: 10.1038/srep07094 (PMC4235284; doi:10.1038/srep07094)
Supplement: Supplementary Information — Supplementary data [file srep07094-s1.pdf]

Development and application of loop-mediated isothermal amplification for detection of the F167Y mutation of benzimidazole-resistant isolates in *Fusarium graminearum*

Yabing Duan, Xiaoke Zhang, Changyan Ge, Yong Wang, Junhong Cao, Xiaojing Jia, Jianxin Wang & Mingguo Zhou
College of Plant Protection, Nanjing Agricultural University, Nanjing, 210095, China

Correspondence and requests for materials should be addressed to M. G. Z. (mgzhou@njau.edu.cn)

Figure legend

Fig. S1 Sensitivity of LAMP vs. conventional PCR for detection of the F167Y mutation. (A) Gel electrophoresis of LAMP. (B) Gel electrophoresis of PCR. Concentrations of template DNA (ng μL^{-1}) per reaction in (A), and (B) were: 1=10^0, 2=10^{-1}, 3=10^{-2}, 4=10^{-3}, 5=10^{-4}, 6=10^{-5}, and 7=10^{-6}. In (A) and (B), M indicates 100-bp, and 250-bp ladder, respectively.

Figure S1

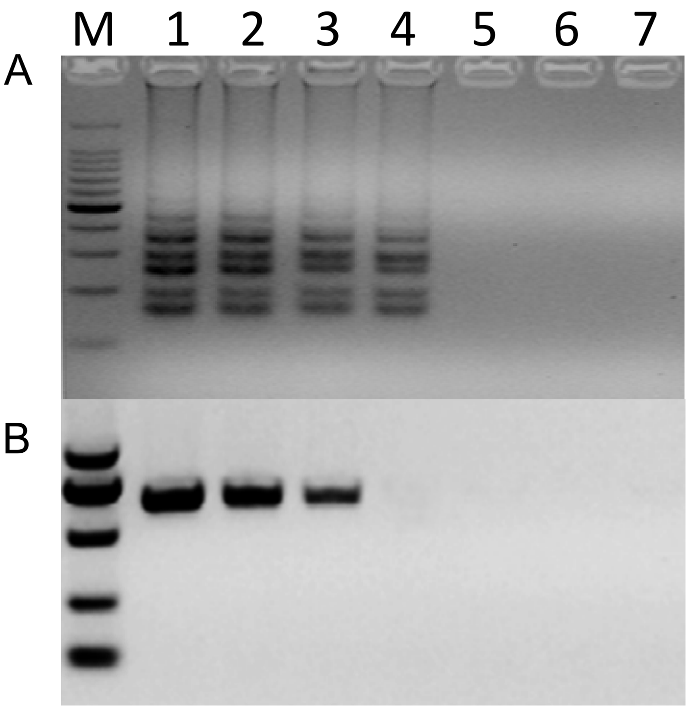

Supplementary Tables

Table S1. Information of the primers used in this study.

Table S2. *Fusarium graminearum* isolates used in specificity test of LAMP.

Table S3. *Fusarium graminearum* isolates used in repeatability test of LAMP

Tables

Table S1

Primers	Sequence (5’-3’)[a]	Use
F3	TTCCAGCTGACGCACTCT	Forward outer primer for LAMP
B3	ACAGAAGGTCTCGTCAGAGT	Backward outer primer for LAMP
FIP	TGCGATCGGGGAACTCCTCG-TGGTACCGGTTCCGGTATG	Forward inner primer for LAMP
BIP1	CTATTCCGTTATGCCCTCGCCC-ACGAGCTGGTTCAGAGACAA	
BIP2	TATTCCGTTATGCCCTCGCCC-ACGAGCTGGTTCAGAGACAA	
BIP3	ATTCCGTTATGCCCTCGCCC-ACGAGCTGGTTCAGAGACAA	Backward inner primers to distinguish the *Fusarium graminearum* genotypes (F167Y) for LAMP
BIP4	GAATTCCGTTATGCCCTCGCCC-ACGAGCTGGTTCAGAGACAA	
BIP5	AATTCCGTTATGCCCTCGCCC-ACGAGCTGGTTCAGAGACAA	
BIP6	AAATTCCGTTATGCCCTCGCCC-ACGAGCTGGTTCAGAGACAA	
BIP7	AAACCGTTATGCCCTCGCCC-ACGAGCTGGTTCAGAGACAA	
Fgbeta637F	TCAGGGCGGCAACGACAAGTA	To amplify the partial fragments (637 bp) of the β_2-tubulin gene containing the F167Y position.
Fgbeta637R	GTGAAGTCGGGGGAACGGAAT	

[a] Nucleotides in frames are modified from the sequence of the β_2-tubulin gene in the sensitive biotype and the resistant biotype. Nucleotides in bold are mismatches manually added specifically to distinguish *F. graminearum* genotypes (F167Y).

Table S2

Isolates	Genotype description	Origin	Resistance phenotype[a]	LAMP[b]
R9	Mutation at codon 167 of β_2 tubulin (F167Y)	Zhejiang province, China	MBC^R	＋
2021	Wild type	Zhejiang province, China	MBC^S	－
Y50C	Mutation at codon 50 of β_2 tubulin (Y50C)	In our Lab.	MBC^R	－
J-2	Mutation at codon 198 of β_2 tubulin (E198K)	Anhui province, China	MBC^R	－
ZJ80	Mutation at codon 198 of β_2 tubulin (E198Q)	Anhui province, China	MBC^R	－
NT-7	Mutation at codon 200 of β_2 tubulin (F200Y)	Hubei province, China	MBC^R	－

[a] MBC^S and MBC^R indicate that the isolate is sensitive and resistant to carbendazim, respectively.

[b] '＋' and '－' represent positive and negative results, respectively.

Table S3

Order	Isolates	Genotype description	Origin	Resistance phenotype[a]	LAMP[b]
1	YZ1001	Mutation at codon 167 of β_2 tubulin (F167Y)	Jiangsu province, China	MBC^R	＋
2	CZ1001	Mutation at codon 167 of β_2 tubulin (F167Y)	Jiangsu province, China	MBC^R	＋
3	LYG1209	Mutation at codon 167 of β_2 tubulin (F167Y)	Jiangsu province, China	MBC^R	＋
4	TZ1205	Mutation at codon 167 of β_2 tubulin (F167Y)	Jiangsu province, China	MBC^R	＋
5	HA1008	Mutation at codon 167 of β_2 tubulin (F167Y)	Jiangsu province, China	MBC^R	＋
6	WX1002	Mutation at codon 167 of β_2 tubulin (F167Y)	Jiangsu province, China	MBC^R	＋
7	YC1011	Mutation at codon 167 of β_2 tubulin (F167Y)	Jiangsu province, China	MBC^R	＋
8	JH1408	Mutation at codon 167 of β_2 tubulin (F167Y)	Jiangsu province, China	MBC^R	＋
9	JH1411	Mutation at codon 167 of β_2 tubulin (F167Y)	Jiangsu province, China	MBC^R	＋
10	JH1463	Mutation at codon 167 of β_2 tubulin (F167Y)	Jiangsu province, China	MBC^R	＋
11	XS1403	Mutation at codon 167 of β_2 tubulin (F167Y)	Jiangsu province, China	MBC^R	＋
12	XS1433	Mutation at codon 167 of β_2 tubulin (F167Y)	Jiangsu province, China	MBC^R	＋
13	XS1443	Mutation at codon 167 of β_2 tubulin (F167Y)	Jiangsu province, China	MBC^R	＋
14	XS1451	Mutation at codon 167 of β_2 tubulin (F167Y)	Jiangsu province, China	MBC^R	＋
15	CHZ1013	Mutation at codon 167 of β_2 tubulin (F167Y)	Anhui province, China	MBC^R	＋
16	SUZ1003	Mutation at codon 167 of β_2 tubulin (F167Y)	Anhui province, China	MBC^R	＋
17	LJ1403	Mutation at codon 167 of β_2 tubulin (F167Y)	Anhui province, China	MBC^R	＋
18	LJ1411	Mutation at codon 167 of β_2 tubulin (F167Y)	Anhui province, China	MBC^R	＋
19	SC1103	Mutation at codon 167 of β_2 tubulin (F167Y)	Sichuan province, China	MBC^R	＋
20	SC1105	Mutation at codon 167 of β_2 tubulin (F167Y)	Sichuan province, China	MBC^R	＋
21	SC1108	Mutation at codon 167 of β_2 tubulin (F167Y)	Sichuan province, China	MBC^R	＋
22	HB1002	Mutation at codon 167 of β_2 tubulin (F167Y)	Hubei province, China	MBC^R	＋
23	HB1003	Mutation at codon 167 of β_2 tubulin (F167Y)	Hubei province, China	MBC^R	＋
24	LY1102	Mutation at codon 167 of β_2 tubulin (F167Y)	Shandong province, China	MBC^R	＋
25	LY1108	Mutation at codon 167 of β_2 tubulin (F167Y)	Shandong province, China	MBC^R	＋
26	LY1110	Mutation at codon 167 of β_2 tubulin (F167Y)	Shandong province, China	MBC^R	＋
27	LH1201	Mutation at codon 167 of β_2 tubulin (F167Y)	Henan province, China	MBC^R	＋
28	LH1001	Mutation at codon 167 of β_2 tubulin (F167Y)	Henan province, China	MBC^R	＋

[a] MBC^R indicate that the isolate is sensitive and resistant to carbendazim, respectively.

[b] '＋'represent positive results.
